# A Cadaveric Study of the Distal Biceps Femoris Muscle in relation to the Normal and Variant Course of the Common Peroneal Nerve: A Possible Cause of Common Peroneal Entrapment Neuropathy

**DOI:** 10.1155/2020/3093874

**Published:** 2020-10-13

**Authors:** Jeong-Hyun Park, Jinseo Yang, Kwang-Rak Park, Tae Woo Kim, Taeyeong Kim, Suyeon Park, Battur Tsengel, Jaeho Cho

**Affiliations:** ^1^Department of Anatomy & Cell Biology, Graduate School of Medicine, Kangwon National University, Kangwon, Republic of Korea; ^2^Department of Neurosurgery, Chuncheon Sacred Heart Hospital, Hallym University, Chuncheon, Republic of Korea; ^3^Department of Orthopaedic Surgery, Seoul Metropolitan Government-Seoul National University, Boramae Medical Center, Seoul, Republic of Korea; ^4^Department of Orthopaedic Surgery, Chuncheon Sacred Heart Hospital, Hallym University, Chuncheon, Republic of Korea; ^5^Department of biostatistics, Soonchunhyang University Hospital, College of medicine, Seoul, Republic of Korea; ^6^Institute for Skeletal Aging and Orthopedic Surgery, Chuncheon Sacred Heart Hospital, Hallym University, Chuncheon, Republic of Korea

## Abstract

The most frequent mononeuropathy in the lower extremity has been reported as the common peroneal nerve entrapment neuropathy (CPNe) around the head and neck of the fibula, although the mechanism of the neuropathy in this area cannot be fully explained. Therefore, the aim of this cadaveric study was to evaluate the relationship between morphologic variations of the distal biceps femoris muscle (BFM) and the course of the common peroneal nerve (CPN) and to investigate the incidence and morphological characteristics of anatomical variations in the BFM associated with CPNe. The popliteal region and the thigh were dissected in 115 formalin-fixed lower limbs. We evaluated consensus for (1) normal anatomy of the distal BFM, (2) anatomic variations of this muscle, and (3) the relationship of the muscle to the CPN. Measurements of the distal extents of the short and long heads of the BFM from insertion (fibular head) were performed. Two anatomic patterns were seen. First, in 93 knees (80.8%), the CPN ran obliquely along the lateral side of the BFM and then superficial to the lateral head of the gastrocnemius muscle. Second, in 22 cases (19.2%), the CPN coursed within a tunnel between the biceps femoris and lateral head of the gastrocnemius muscle (LGCM). There was a positive correlation between the distal extents of the short heads of the biceps femoris muscle (SHBFM) and the presence of the tunnel. The “popliteal intermuscular tunnel” in which the CPN travels can be produced between the more distal extension variant of the SHBFM and the LGCM. This anatomical variation of BFM may have a clinical significance as an entrapment area of the CPN in the patients in which the mechanism of CPNe around the fibula head and neck is not understood.

## 1. Introduction

The common peroneal entrapment neuropathy (CPNe) is the most common lower extremity entrapment neuropathy and accounts for 15% of all peripheral entrapment neuropathies [1, 2]. The common peroneal nerve (CPN) is an important nerve to consider when performing a complete neurologic evaluation in the lower extremity, because it can elicit a host of problems if damaged, including loss of sensation or paresthesia over the anterior leg and dorsum of the foot. Moreover, if severely damaged, it can also affect motor function causing gait disturbances, such as foot drop, which have a significant impact on a patient's quality of life [3, 4].

The CPN branches from the sciatic nerve, usually arising at the junction of the upper two-thirds and lower third of the posterior compartment of the thigh. It descends obliquely along the posterolateral side of the popliteal fossa to the head of the fibula. It courses close to the medial margin of the biceps femoris muscle (BFM) and lies between the tendon and lateral head of the gastrocnemius muscle (LGCM), and then winds around the neck of the fibula through an area known as the fibular tunnel [5–8]. In the reviewed literature, most authors have reported that CPNe occurs around the fibular head and neck, due to the fact that it is superficial in the subcutaneous space directly over the unyielding fibula and is tethered by tight fascial bands [1, 3, 9–12]. Although the mechanism by which the CPN is compressed in the fibular tunnel could not be clearly explained by any of the researchers, this anatomic location was believed to cause a significant compression of the CPN [8].

Vieira et al. [13] proposed the possibility of anatomical variation of the distal BFM related to the CPNe. These authors suggested investigating the course of the CPN between popliteal muscles, including the short head of the biceps femoris muscle (SHBFM) and the LGCM, using magnetic resonance imaging (MRI). We also reviewed an MRI study about an anatomical variation of the distal BFM related to the CPNe with a clinical case illustration [14]. However, due to limitations of MRI-based imaging studies, these studies have failed to completely establish morphological characteristics of BFM variation-related entrapment neuropathy. To overcome this problem, an anatomical study using cadavers was required.

The aim of this cadaveric study was to evaluate the relationship between variations of the distal BFM and the course of the CPN. We then investigated the incidence and morphological characteristics of anatomical variation in the BFM associated with CPNe and discussed its clinical significance.

## 2. Materials and Methods

The cadavers used in the present study were donated to the University of Medicine with consent for education and research. In addition, this study was approved by the Ethics Committee of our institution (Chuncheon Sacred Heart Hospital, Hallym University, NON2019-006), as a cadaveric study.

One hundred fifteen (61 left and 54 right) lower limb specimens in formalin-fixed adult cadavers were dissected. Of the 115 specimens, 47 (40.9%) were from female and 68 (59.1%) from male cadavers. The mean age of the donors at death was 75.7 (SD 12.3, median 78 and range 36-94) years. The popliteal regions and the thighs of all cadavers demonstrated intact skin and no signs of previous trauma or surgery, obvious deformities, or ulcers.

The cadaver specimens were stabilized in the prone position, and the routine dissection of the popliteal area at the knee joint level was conducted on each one. A section of the skin was meticulously dissected, and the diamond-shaped popliteal fossa was exposed with four borders, consisting of the semimembranosus muscle, the BFM, medial head of the gastrocnemius muscle (MGCM), and the LGCM.

We evaluated consensus for (1) normal anatomy of the distal BFM, (2) anatomic variations of this muscle, and (3) the relationship of the muscle to the CPN. The subjects were divided into two groups (typical group; Type I and variant group; Type II) according to the anatomical course of the CPN associated with the distal BFM.

**Figure 1:**
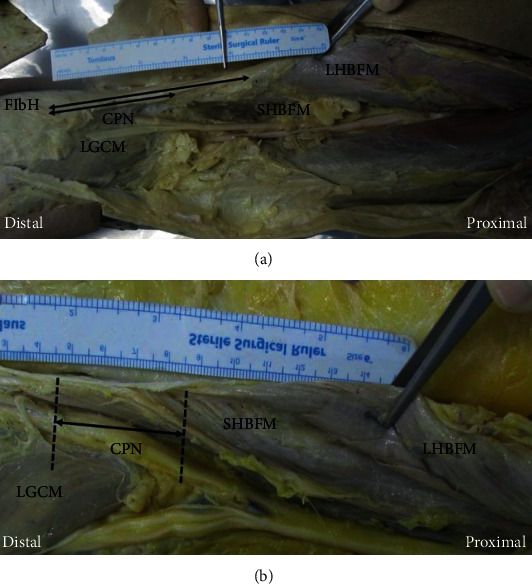
(a) The measurement of the length between the fibular head and musculotendinous junction of BFM. (b) The measurement of the length of popliteal intermuscular tunnel. CPN: common peroneal nerve; FibH: fibular head; LGCM: lateral head of the gastrocnemius muscle; LHBFM: long head of the biceps femoris muscle; SHBFM = short head of the biceps femoris muscle.

To evaluate quantitative morphology of the BFM, we measured the length between the fibular head and the musculotendinous junction of the BFM (Figure 1(a)). There were various differences in the extent of the tendon attachment of the BFM to the fibular head. Thus, in all cases, the length was uniformly measured based on the most proximal end of the fibular head. In the variant group with tunnel formation, the length of the popliteal intermuscular tunnel was measured (Figure 1(b)). The averages of the two researchers' measurements were recorded to describe each specimen.

Inter- and intraobserver reliabilities were obtained for all measurements using the intraclass correlation coefficient (ICC). According to the definitions of Landis and Koch [15], ICCs of 0.81 to 1.00, 0.61 to 0.80, 0.41 to 0.60, 0.21 to 0.40, and 0.00 to 0.20 were interpreted as excellent, good, moderate, fair, and poor, respectively. The Wilcoxon–Mann–Whitney test and chi-squared test were performed to compare the measurements between the Type I and Type II groups. Receiver operating statistics (ROC) analysis was performed to find the best value of measurements of the BFM variant to predict whether the popliteal tunnel existed and was associated with the course of the CPN. Resulting optimized sensitivity and specificity were calculated. All statistical analyses were performed using the SPSS Statistics for Windows, version 24.0 (IBM Corp. Armonk, NY). A *p* value less than 0.05 was considered statistically significant.

## 3. Results

Intraclass correlation coefficients were generated for all measurements. All measurements were higher than 0.80 (indicating acceptable reliability) and were employed in the study.

**Figure 2:**
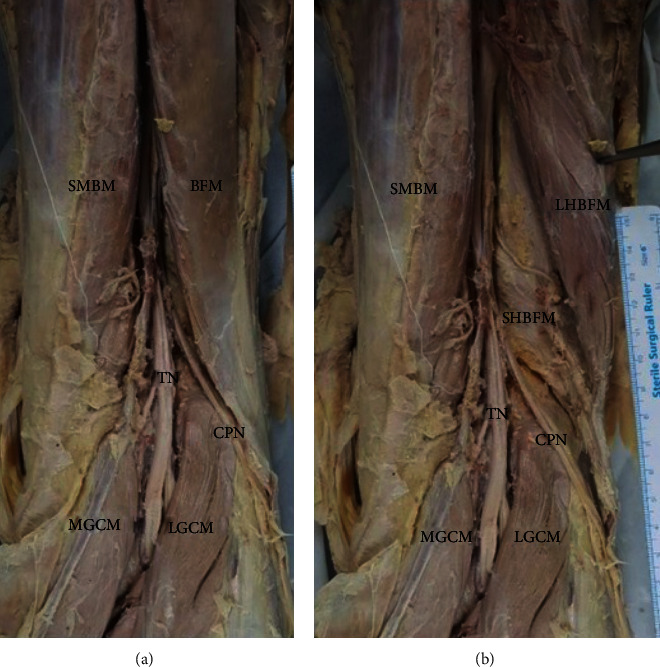
(a) Typically, the common peroneal nerve runs obliquely along the medial side of the biceps femoris muscle and then (b) superficially to the lateral head of the gastrocnemius muscle. BFM: biceps femoris muscle; CPN: common peroneal nerve; LGCM: lateral head of the gastrocnemius muscle; MGCM: medial head of the gastrocnemius muscle; SMBM: semimembranosus muscle; TN: tibial nerve.

**Figure 3:**
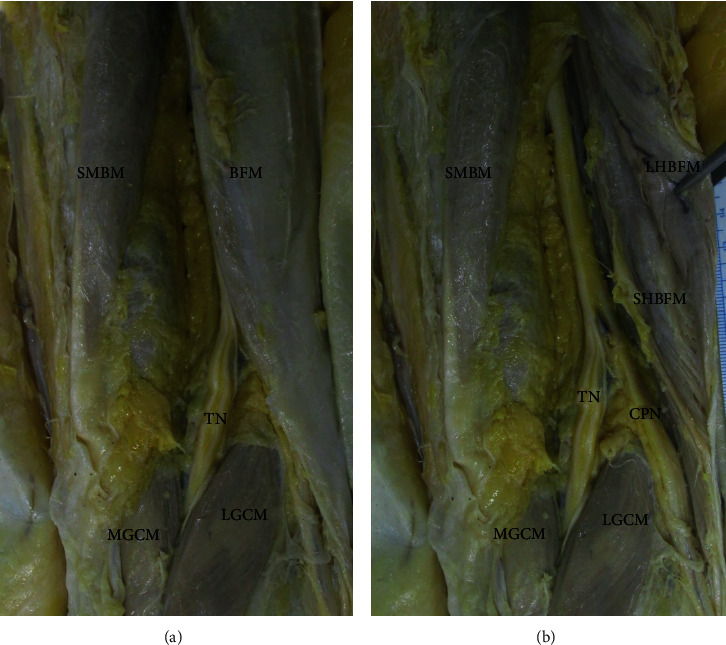
(a) The common peroneal nerve is not seen at the medial margin of the popliteal fossa. (b) The common peroneal nerve runs under the cover of the biceps femoris muscle and then descends below the muscle. The short head of the biceps femoris muscle was extended more distal and posteriorly. The common peroneal nerve runs within tunnel formed between the lateral head of the gastrocnemius muscle and the short head of the biceps femoris muscle. CPN: common peroneal nerve; LGCM: lateral head of the gastrocnemius muscle; LHBFM: long head of the biceps femoris muscle; MGCM: medial head of the gastrocnemius muscle; SHBFM: short head of the biceps femoris muscle; SMBM: semimembranosus muscle; TN: tibial nerve.

Typically, the CPN arises from the sciatic nerve at the upper level of the popliteal fossa and runs obliquely along the medial side of the BFM and then superficial to the LGCM (Figure 2). The authors reported a cadaveric case with variant of distal BFM associated with CPNe [16]. In cadavers with variation, the tibial nerve was found at the popliteal fossa, but the CPN was not observed at its medial margin. The distal BFM was identified and then reflected to examine its deep layer. The CPN descended below the long head of the BFM and along the SHBFM, which was extended more distally and posteriorly. More inferiorly, due to the variation in the muscular structure, the CPN was situated within a tunnel in which the floor was created by the by the LGCM and the roof was created by the SHBFM (Figure 3).

**Figure 4:**
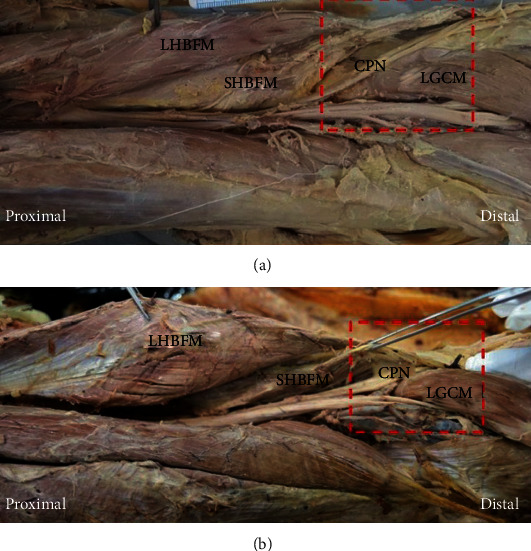
Types according to the anatomical course of the CPN associated with the tunnel between the SHBFM and the LGCM (red dotted-line): (a) Type I and (b) Type II. CPN: common peroneal nerve; LGCM: lateral head of the gastrocnemius muscle; LHBFM: long head of the biceps femoris muscle; SHBFM: short head of the biceps femoris muscle.

Thus, two anatomic patterns were seen: (1) Type I (typical type) was observed in 93 specimens (80.8%), in which the CPN runs obliquely along the medial side of the BFM and then superficial to the LGCM; and (2) Type II (variant type), in 22 specimens (19.2%), in which the CPN traversed within a narrow fatty tunnel between the BFM and LGCM (Figure 4).

**Figure 5:**
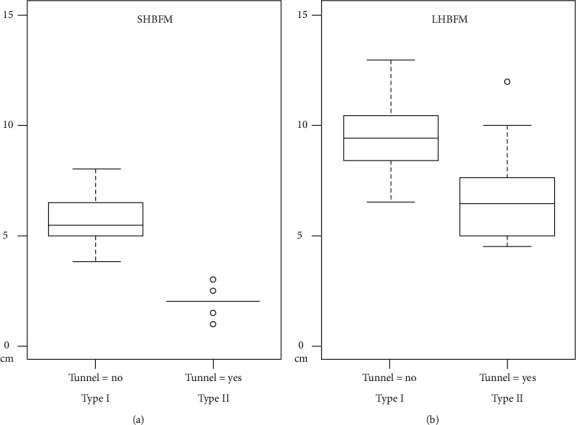
Comparison of the distal extents of the biceps femoris muscle between Type I and II. (a) Short head of the biceps femoris muscle (SHBFM). (b) Long head of the biceps femoris muscle (LHBPM). Both, *p* < 0.001.

There was no significant difference by age (Mann–Whitney test: *p* = 0.419). The distribution of anatomic patterns showed no significant difference by sex or side (Table 1). The length between the fibular head and the musculotendinous junction of LHBFM was 9.4 cm (SD 1.5) in Type I, while it was 6.7 cm (SD 1.9) in Type II. Also, the length between the fibular head and the musculotendinous junction of the SHBFM was 5.6 cm (SD 1.1) in Type I, while it was 2.0 cm (SD 0.6) in Type II. There was a significant correlation in the distal extents of the BFM according to the presence of the tunnel (Figure 5).

All subjects in the Type II group had an average tunnel length of 3.07 cm (range 2.3 to 4.4 cm). Concerning the prediction presence of the tunnel by ROC, distal extension of the SHBFM with best sensitivity and specificity had a cutoff value of <3.4 cm from the fibula head, reaching a sensitivity of 100% and a specificity of 100%, and distal extension of the LHBFM with best sensitivity and specificity had a cutoff value of <8.2 cm from the fibular head, reaching a sensitivity of 91% and a specificity of 75%.

## 4. Discussion

The present cadaveric study describes the morphologic variation of the distal BFM and association of this muscle with the course of the CPN. The CPN typically descends posteriorly to the SHBFM and then superficially to the LGCM. However, about 20% of the time, the BFM (especially the SHBFM) was extended more distally and posteriorly. As this muscle variant existed as a roof-like structure for the CPN, the CPN passed through a tunnel formed between the SHBFM and the LGCM. Therefore, the presence of a popliteal intermuscular tunnel can be assumed to be a possible entrapment area of the CPN.

In the literature review, the anatomical location of the CPNe is known as the fibular head. The compression of the CPN at the fibular head has been described as an entrapment in the tunnel created by the two peroneus longus muscles [12]. Dellen et al. [17] reported that the mean length of the fibrous band of the fibular tunnel was 9.1 mm in cadavers and 10.1 mm in patients. In our study, the average length of the popliteal tunnel formed by the SHBFM and the LGCM was 3.07 cm (range 2.3 to 4.4 cm). Also, the length of the popliteal intermuscular tunnel in symptomatic patients with anatomical variation of the distal BFM related to the CPNe was 24 mm, which was confirmed by MRI [14]. Therefore, compared to the length of the fibula tunnel, the length of the popliteal tunnel is sufficient for the nerve to be compressed, so it is thought that this tunnel can be a predisposing clinical factor in CPNe.

The posterior compartment contains the three hamstring muscles: biceps femoris, semimembranosus, and semitendinosus. Textbooks related to anatomical variation describe that the distal tendons of all the hamstring muscles display a large amount of variation in the tendinous band, not muscle itself. Also, most of the tendon variation relates to the semitendinosus tendon [18]. Although a rare third head of the BFM related to the tenuissimus, a phylogenetic remamant, was reported [19], the BFM has two heads of origin typically. The long head arises from the ischial tuberosity, while the short head arises from the lateral prolongation of the linea aspera of the femur. The tendons of the short head and long head merge above the knee joint, and the combined tendon inserts into the head of the fibula [20]. According to a three-dimensional anatomical study of the tendon-bone junctions of the knee joint posterolateral complex [21], both the area and the center of the insertion site of the BFM have racial differences. One study observed that the musculotendinous junction of the LHBFM is 7-10 cm above the knee joint level, but no information has been found on the SHBFM. The present study suggested that the distance of the musculotendinous junction of the LHBFM from insertion (fibular head) was 9.4 cm on average and that of the SHBFM was 5.6 cm on average. The tunnel formed with regard to the CPNe was found to be related to the distal extent by the variation of the LHBFM as well as variation of the SHBFM. In particular, the variation in the SHBFM was significantly correlated to the formation of the tunnel, and it was statistically postulated that the tunnel could exist when the SHBFM descended distally within 3.4 cm of the insertion. Thus, our research provides valuable data and knowledge concerning morphologic variations of the BFM as a potential risk factor of CPNe.

In the reviewed literature, most authors have reported that CPNe occurs around the fibular head and neck [1, 3, 9–12]. In the human fetuses, the terminal branch of CPN was based on the head of the fibula: (i) high cleavage-above the head of the fibula (1%), (ii) median cleavage-fibula head height (34%), and (iii) low cleavage-below the fibula head (65%) [22]. Also, CPN is superficial in the subcutaneous space directly over the unyielding fibula and is tethered by tight fascial bands. In order to recompress this condition, studies on the thread CPN release method as well as the open method have been introduced [3, 23]. However, the mechanism by which the CPN is compressed in the fibular tunnel could not be clearly explained by any of the researchers; this anatomic location was believed to cause significant compression of the CPN [8].

Posture-induced CPNe could occur after maintaining a certain posture for a long time. The kneeling and squatting postures most often induced CPNe in Asians [24]. Masakado et al. [8] defined the fibular tunnel as a compression site of the CPN when the knee was hyperflexed, but these authors could not accurately explain the mechanism for entrapment of the CPN. The CPN could be compressed at the popliteal tunnel formed by variant of the distal BFM rather than the fibular tunnel, because the popliteal fossa would be affected by the hyperflexion of the knee during kneeling and squatting postures. Therefore, we suggest that the variant of distal BFM may be considered clinically as a possible entrapment factor, if the mechanism of the neuropathy cannot be fully explained by the fibular head, such as in the case of posture-induced neuropathy.

There are limitations in this study. Anatomical variation of the CPN itself may occur but is not considered in this study. For instance, the level of the sciatic nerve divided into the CPN and the TN may vary and various patterns of division in the CPN with respect to the superficial and deep branches have been described below the level of the knee joint more distally than the popliteal intermuscular tunnel in most cadaveric studies [5, 6]. Also, we did not evaluate the contribution of the LGCM muscle to the formation of the popliteal intermuscular tunnel. However, we believe that the variations in the gastrocnemius muscle did not contribute significantly to tunnel formation, because we found very few anatomic variations of the gastrocnemius muscle during cadaveric dissection.

## 5. Conclusions

The common peroneal nerve courses through the “popliteal intermuscular tunnel” formed between the more posterior or distal extension of the SHBFM and the LGCM in about 20% of the population. This knowledge that anatomical variation of the distal BFM is a potential factor of CPNe can be used for more accurate diagnosis in the patients in which CPNe around the fibula head and neck is not well-understood. Also, this anatomical data may be clinically useful to treat CPNe associated with variation of the distal BFM.

## Figures and Tables

**Table 1:** The distribution of anatomic patterns of BFM by sex and side.

	Type		*p* value^₭^
	I	II	
Sex			
*Male*			
*n*	53	15	0.337
%	57.0%	68.2%	
*Female*			
*n*	40	7	
%	43.0%	31.8%	
Side			
Right			
*n*	43	11	0.750
%	46.2%	50.0%	
Left			
*n*	50	11	
%	53.8%	50.0%	
^₭^ *p* value by chi-squared test	93(80.8%)	22(19.2%)	

## Data Availability

The data used to support the findings of this study are available from the corresponding author upon request.

## Abbreviation

CPN: Common peroneal nerve
CPNe: Common peroneal nerve entrapment neuropathy
LGCM: Lateral head of the gastrocnemius muscle
LHBFM: Long head of the biceps femoris muscle
MRI: Magnetic resonance imaging
SHBFM: Short head of the biceps femoris muscle.
